# Dual leucine zipper kinase regulates expression of axon guidance genes in mouse neuronal cells

**DOI:** 10.1186/s13064-016-0068-8

**Published:** 2016-07-28

**Authors:** Andréanne Blondeau, Jean-François Lucier, Dominick Matteau, Lauralyne Dumont, Sébastien Rodrigue, Pierre-Étienne Jacques, Richard Blouin

**Affiliations:** 1 Département de biologie, Faculté des sciences, Université de Sherbrooke, 2500 Boul. de l’Université, Sherbrooke, Québec J1K 2R1 Canada; 2 Département d’informatique, Faculté des sciences, Université de Sherbrooke, Sherbrooke, Québec Canada; 3 Centre de recherche du Centre hospitalier universitaire de Sherbrooke, Sherbrooke, Canada

**Keywords:** DLK, Neurons, Axon guidance

## Abstract

**Background:**

Recent genetic studies in model organisms, such as *Drosophila*, *C. elegans* and mice, have highlighted a critical role for dual leucine zipper kinase (DLK) in neural development and axonal responses to injury. However, exactly how DLK fulfills these functions remains to be determined. Using RNA-seq profiling, we evaluated the global changes in gene expression that are caused by shRNA-mediated knockdown of endogenous DLK in differentiated Neuro-2a neuroblastoma cells.

**Results:**

Our analysis led to the identification of numerous up- and down-regulated genes, among which several were found to be associated with system development and axon guidance according to gene ontology (GO) and Kyoto Encyclopedia of Genes and Genomes (KEGG) pathway analyses, respectively. Because of their importance in axonal growth, pruning and regeneration during development and adult life, we then examined by quantitative RT-PCR the mRNA expression levels of the identified axon guidance genes in DLK-depleted cells. Consistent with the RNA-seq data, our results confirmed that loss of DLK altered expression of the genes encoding neuropilin 1 (Nrp1), plexin A4 (Plxna4), Eph receptor A7 (Epha7), Rho family GTPase 1 (Rnd1) and semaphorin 6B (Sema6b). Interestingly, this regulation of Nrp1 and Plxna4 mRNA expression by DLK in Neuro-2a cells was also reflected at the protein level, implicating DLK in the modulation of the function of these axon guidance molecules.

**Conclusions:**

Collectively, these results provide the first evidence that axon guidance genes are downstream targets of the DLK signaling pathway, which through their regulation probably modulates neuronal cell development, structure and function.

**Electronic supplementary material:**

The online version of this article (doi:10.1186/s13064-016-0068-8) contains supplementary material, which is available to authorized users.

## Background

The mitogen-activated protein kinases (MAPKs) are important regulators of fundamental biological processes, such as cell proliferation, differentiation, cell survival, migration and apoptosis. These enzymes are activated in response to extracellular stimuli by upstream kinases termed MAPKKs, which are themselves activated by the third component of the MAPK system, the MAPKKKs [1]. Based on their structural and biochemical features, three major subgroups of MAPKs have been described in mammals, including extracellular signal-regulated kinases (ERKs), p38 kinases, and c-Jun N-terminal kinases (JNKs) [1, 2].

One MAPKKK that emerges as a pivotal component of the MAPK pathways is dual leucine zipper kinase (DLK, also known as Map3k12), which was originally identified in a screen for proteins differentially expressed during the retinoic acid-induced differentiation of human NT2 teratocarcinoma cells [3]. DLK preferentially activates JNK, although a role for DLK in activation of ERK and p38 MAPKs has also been proposed [4–6]. Based on Northern blot analysis, *in situ* hybridization and immunolocalization, DLK has a tissue-specific expression pattern in both mouse and human, being mainly expressed in brain, kidney and skin [3, 7–10]. Previous studies have suggested a fundamental role for DLK in vivo since targeted deletion of the *DLK* gene in mice results in perinatal death [11]. Embryos lacking *DLK* display abnormal brain development, characterized by defects in axon growth, neuron migration, apoptosis and axon degeneration [11–15]. Apart from its role during development, DLK has also been shown to regulate axonal damage signaling in mature neurons [13, 15–17]. For instance, as demonstrated by studies in mice and rats, loss of DLK protects neurons from somal and axonal degeneration in response to mechanical injury, growth factor deprivation and glutamate-induced excitotoxicity [16–19]. Recently, it was also discovered that DLK is required for axonal regeneration in adult peripheral nerves after axotomy in both vertebrate and invertebrate organisms [16, 20, 21]. These findings demonstrate a key role for DLK in controlling neuronal development as well as degenerative and regenerative responses to axonal injury. Although JNK activation is an important and established event downstream of DLK, precisely how DLK mediates such diverse effects in neurons remains an open question.

Because one way to unravel the mode of action of DLK is to identify genes critical for its function in neurons, we characterized by next-generation sequencing (RNA-seq) the transcriptome of differentiated Neuro-2a neuroblastoma cells in which DLK has been depleted by RNA interference. Our results led to the identification of many genes whose expression was significantly altered upon DLK knockdown. Notably, among the identified genes, we focused on those encoding axon guidance molecules due to their crucial roles in many aspects of neuronal development, including axon pathfinding, axon growth, neuronal polarization, neuronal migration and dendrite formation, as well as in axon regeneration in the adult nervous system [22–25].

## Methods

### Antibodies

The polyclonal antiserum used for detection of DLK was described previously [26]. The polyclonal or monoclonal antibodies against phospho-JNK (Thr183/Tyr185, #4671), JNK (#9252), phospho-c-Jun (Ser63, #9261), Nrp1 (#3725) and Plxna4 (#3816) were purchased from Cell Signaling Technology, Inc. (Danvers, MA). The polyclonal antibody against γ-actin (#NB600-533) was from Novus Biologicals (Oakville, Ontario).

### Cell culture

Mouse Neuro-2a neuroblastoma cells were grown in Dulbecco’s modified Eagle’s medium (DMEM) supplemented with 10 % (v/v) fetal bovine serum (FBS), 100 U/ml penicillin and 100 μg/ml streptomycin. When indicated, cells were differentiated by incubation in DMEM containing 0.1 % bovine serum albumin for 24 h.

### Lentivirus production and infection of Neuro-2a cells

HEK 293T cells grown in DMEM supplemented with 10 % (v/v) FBS and antibiotics were cotransfected with the envelope protein expressing vector pMD2.G and the packaging protein expressing vector psPAX2, (kindly provided by Dr. Didier Trono University of Geneva Medical School, Geneva, Switzerland) and with either the transfer pLKO.1 empty lentiviral vector [27] (Addgene, Cambridge, MA, USA, plasmid 8453) or the pLKO.1-based lentiviral mouse DLK shRNA vector (clone TRCN0000022573 [sh73] or clone TRCN0000022569 [sh69], Open Biosystems, Huntsville, AL, USA) using Polyethylenimine Max (#24765, Polysciences Warrington, PA). At 72 h post-transfection, the culture medium containing lentiviruses was harvested, filtered through 0.45-μm filter, and used for infection. Neuro-2a cells, seeded at a density of 0.3 × 10^6^ cells per well in six-well dishes, 0.5 × 10^6^ cells in 60-mm dishes or 2.0 × 10^6^ cells in 100-mm dishes 24 h before, were infected with viral supernatants supplemented with 8 μg/ml polybrene. Two days later, infected cells were treated with puromycin (2.5 μg/ml) and selected for 2 days, after which they were induced to differentiate as mentioned above.

### qRT-PCR experiments

Total RNA was extracted with the Direct-zol RNA MiniPrep kit (#R2050, Zymo Research) in combination with TRIzol (#15596-026, Life Technologies), following the manufacturer’s protocol. A 30 min on-column DNase treatment was performed before elution according to manufacturer’s instructions. RNA was quantified on a NanoDrop (Thermo Scientific) spectrophotometer. Total RNA quality was assessed with an Agilent 2100 Bioanalyzer (Agilent Technologies). Reverse transcription was performed on 2.2 μg total RNA with Transcriptor reverse transcriptase, random hexamers, dNTPs (Roche Diagnostics), and 10 units of RNAseOUT (Invitrogen) following the manufacturer’s protocol in a total volume of 20 μl. All forward and reverse primers were individually resuspended to 20–100 μM stock solution in Tris-EDTA buffer (IDT) and diluted as a primer pair to 1 μM in RNase DNase-free water (IDT). Quantitative PCR (qPCR) reactions were performed in 10 μl in 96 well plates on a CFX-96 thermocycler (BioRad) with 5 μL of 2X iTaq Universal SYBR Green Supermix (BioRad), 10 ng (3 μl) cDNA, and 200 nM final (2 μl) primer pair solutions. The following cycling conditions were used: 3 min at 95 °C; 50 cycles: 15 s at 95 °C, 30 s at 60 °C, 30 s at 72 °C. Relative expression levels were calculated using the qBASE framework [28] and the housekeeping genes *Sdha*, *Txnl4b* and *Pum1* for mouse cDNA. Primer design and validation was evaluated as described elsewhere [29]. In every qPCR run, a no-template control was performed for each primer pair and these were consistently negative. All primer sequences are available in Additional file 1: Table S1.

### Imaging and quantification

Images of control and DLK-depleted Neuro-2a cells were acquired in bright-field using a Zeiss AxioObserver.Z1 inverted microscope equipped with a 20x/0.30 NA objective and a Zeiss Axiocam 506 camera. The length of approximately 200 neurites was measured from five random microscope fields per sample using the open source software Fiji [30] and the Simple Neurite Tracer plugin [31]. GraphPad Prism (GraphPad Software Inc, La Jolla, CA, USA) was used to calculate and plot mean and standard error of the mean (SEM) of the measured neurite lengths. Neurites were also classified into five groups based on their length, and their frequency within each group was calculated for both the control and DLK-depleted cells. The mean ± SEM of the frequency of distribution within each group of neurites was determined using GraphPad Prism and presented in percentage. Significance of the results was assessed by Student’s *t* test.

### RNA-seq sample preparation and analysis

Total RNA was isolated as described above from control, sh73/DLK- and sh69/DLK-depleted cells. The quality of total RNA was evaluated using the Agilent 2100 BioAnalyzer and the Agilent RNA 6000 Nano Kit (#5067-1511) according to the manufacturer’s instructions. For all samples, RNA integrity numbers were sufficiently high (>9.5) to perform mRNA sequencing. mRNA was purified from 5 μg of total RNA using the NEBNext Poly(A) mRNA Magnetic Isolation Module (#E7490, New England Biolabs, Whitby, Ontario) following manufacturer’s instructions. cDNA libraries were then prepared with 25 ng mRNA of each sample using the NEBNext Ultra Directional RNA Library Prep Kit for Illumina (#E7420, New England Biolabs) and barcoded by PCR for subsequent multiplexed sequencing. The quality and quantity of the librairies were assessed using the Agilent High Sensitivity DNA Kit (#5067-4626) on Agilent 2100 BioAnalyzer. Sequencing of the multiplexed cDNA libraries was performed on an Illumina HiSeq 2500 system (Illumina, San Diego, CA, USA) in two lanes at the Institut de Recherches Cliniques de Montréal (IRCM). The obtained raw reads were processed using the McGill University and Génome Québec Innovation Centre (MUGQIC) RNA-seq pipeline version 1.4 (https://bitbucket.org/mugqic/mugqic_pipelines). Briefly, the reads were trimmed using Trimmomatic version 0.32 [32] to remove adapter sequences and low quality reads (Phred quality < 30 and minimum lenght of 32), and aligned onto the mouse reference genome (mm10 assembly) using TopHat version 2.0.11 and Bowtie version 2.2.2 [33, 34]. Read counts for each gene from the GRCm38.73 mouse assembly from the Ensembl database [35] were obtained using HTSeq version 0.6.1 [36]. Differential gene expression analyses between control and DLK-depleted cells were performed using DESeq version 1.16 [37] and edgeR version 3.6.8 [38] from the Bioconductor package version 2.14 [39, 40] of R version 3.1.1. The identified genes were further filtered against the following criteria: (i) a minimum of 50 reads per gene, (ii) a fold-change threshold ≥ 2 between control and DLK-depleted cells, and (iii) transcripts altered in the same direction with both shDLK constructs and common to at least three of the four samples of DLK-depleted cells. Functional category and pathway analyses of the differentially expressed genes were performed using DAVID [41].

### Statistical analysis

qRT-PCR and immunoblot data represent the mean ± SEM of at least three independently performed experiments. The statistical significance between mean values was determined by unpaired *t* test with Welch’s correction (two tails) using GraphPad Prism software. p-values of < 0.05 were considered to be statistically signifiant.

## Results

### Depletion of DLK by RNA interference in Neuro-2a cells

**Fig. 1:**
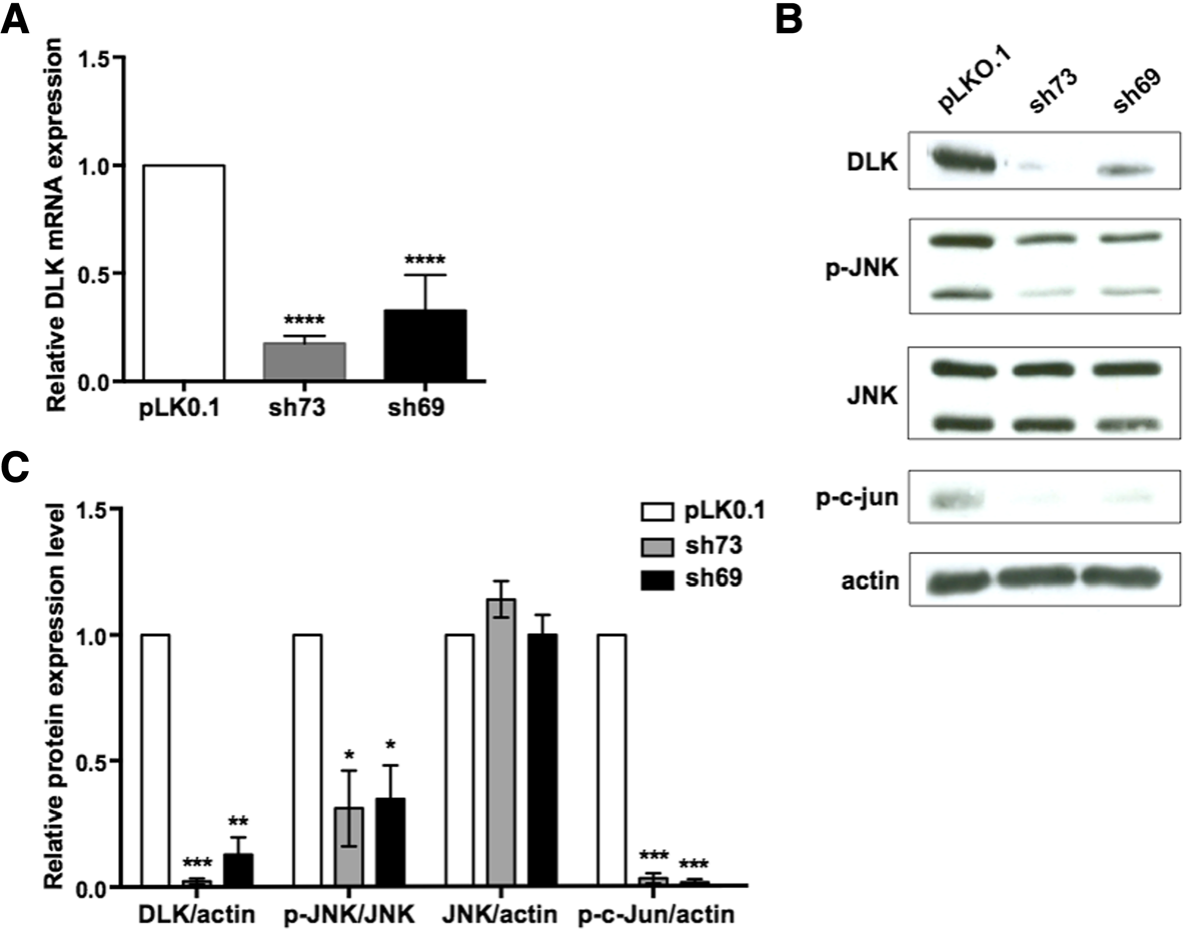
Knockdown of DLK in differentiated Neuro-2a cells. Neuro-2a cells were infected with an empty lentiviral vector (pLKO.1) or with lentivirus expressing mouse DLK shRNAs (sh73 and sh69). After infection and selection with puromycin, cells were subjected to differentiation for 24 h before being processed for total RNA extraction and whole-cell extracts. **a** The relative mRNA level of DLK in infected cells was analyzed by quantitative RT-PCR, normalized to three housekeeping genes and calculated with the ΔΔ*C*_*T*_ method. The value of DLK mRNA expression in control cells (pLKO.1) was arbitrarily set to 1. Data are the mean ± SEM (error bars) from three independent experiments carried out in triplicate. ****, *p* < 0.0001. **b** Representative Western blots showing levels of DLK, phospho-JNK (p-JNK), total JNK, phospho-c-Jun (p-c-Jun) and actin in infected Neuro-2a cells. **c** Quantitative densitometric measurements of DLK, p-JNK, total JNK and p-c-Jun protein levels in infected cells. Results are normalized to either actin or total JNK level in control cells, which were set to 1, and represent mean ± SEM (error bars) from three independent experiments. *, *p* < 0.05; **, *p* < 0.01; ***, *p* < 0.001

Neuro-2a is an established mouse neural crest-derived cell line that has been used extensively for studying neuronal differentiation, axon growth and signaling pathways [42]. Upon treatment with differentiation agents, such as serum-free media, they stop proliferating and show ultrastructural, morphological and functional properties of neurons [43]. In this study, we took advantage of this model system to identify genes downstream of DLK in neuronal cells. To do so, we first silenced the expression of endogenous DLK by infecting Neuro-2a cells with lentiviral vectors expressing two different short hairpin RNAs (shRNA) that target mouse DLK mRNA (sh73 and sh69). To exclude potential nonspecific effects, cells were also infected with an empty lentiviral vector (pLKO.1). After selection with puromycin for 2 days, the infected cells were then induced to differentiate into a neuronal phenotype by replacing the proliferation medium to DMEM with 0.1 % bovine serum albumin (BSA) for 24 h. Silencing of DLK in differentiated Neuro-2a cells was confirmed by both quantitative reverse transcription PCR (qRT-PCR) and immunoblot analysis. As depicted in Fig. 1a, expression of sh73 and sh69 resulted in an overall decrease of DLK transcripts by approximately 70 to 80 % when compared to control cells. Consistent with the extent of mRNA reduction, DLK protein expression dropped by nearly 90 % in cells infected with the DLK shRNA constructs relative to pLKO.1, although the sh73 construct gave a better silencing effect than the sh69 vector (Fig. 1b and c). Importantly, none of these lentiviral vectors altered the intracellular levels of actin and JNK, thereby supporting their specificity.

Since DLK is an upstream activator of the JNK signaling pathway [4], we tested in parallel whether its knockdown by RNA interference in Neuro-2a cells would perturb the activity of JNK and c-Jun, a downstream target. The impact of DLK depletion on JNK signaling was assayed by Western blotting with antibodies specific to the phosphorylated, activated forms of JNK and c-Jun. Interestingly, loss of DLK attenuated by 70–95 % basal JNK or c-Jun activity (Fig. 1b and c), a result reminiscent of the effect of DLK gene disruption in mouse brain [11]. Thus, these results indicate that DLK is required for basal activity of JNK and c-Jun in differentiated Neuro-2a cells.

### Effects of DLK depletion on neurite outgrowth in Neuro-2a cells

**Fig. 2:**
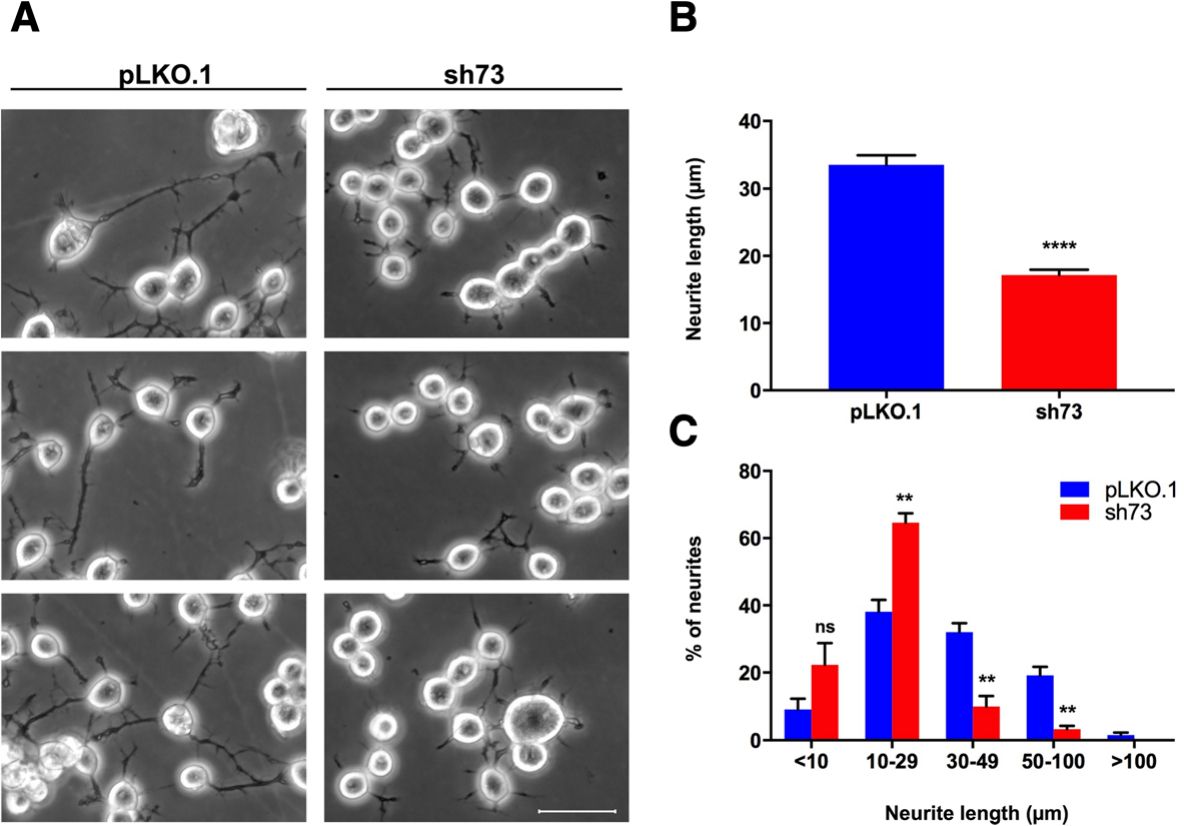
Depletion of DLK in Neuro-2a cells impairs neurite formation. **a** Representative phase contrast micrographs of control (pLKO.1) and DLK-depleted (sh73) Neuro-2a cells induced to differentiate for 24 h. Scale bar, 50 μm. **b** Neurite length of control and DLK-depleted Neuro-2a cells after differentiation. Values represent the mean length of neurites ± SEM (error bars) measured in five randomly chosen microscope fields for each sample (>200 neurites/experimental condition). Statistical significance was determined by unpaired Student’s *t* test. ****, *p* < 0.0001 compared with control cells. **c** Distribution of neurite lengths in control and DLK-depleted Neuro-2a cells after differentiation. Results are expressed as percentage of neurites ± SEM (error bars) with neurite length in the specified range. Statistical significance in neurite length between control and DLK-depleted cells was determined by the multiple *t* test using the Holm-Sidak method with α = 0.05 %. **, *p* < 0.01; ns, *p* > 0.05 vs control

Previous studies have shown that DLK is required for axon outgrowth both in vivo and in vitro [11, 14, 44]. To determine whether this is also the case in our model, we examined the morphology of DLK-depleted Neuro-2a cells, cultured for 24 h in differentiation conditions. DLK depletion in this experiment was done with the sh73 lentiviral vector only because of its better gene silencing efficacy. Representative results of light microscopy demonstrated that DLK-depleted cells had shorter neurites relative to their control counterparts (Fig. 2a), suggesting that neurite outgrowth is inhibited in the absence of DLK. In support of this, we found that there was a 50 % decrease in the average neurite length after DLK knockdown (Fig. 2b). Moreover, analysis of the distribution of neurite lengths indicated that 53 % of neurites in control cells were longer than 30 μm, whereas only 13 % of neurites in DLK-depleted cells were of this size (Fig. 2c). Taken together, these results show that DLK contributes to neurite outgrowth in Neuro-2a cells.

### RNA-seq analysis of differentially expressed genes in DLK-depleted Neuro-2a cells

**Fig. 3:**
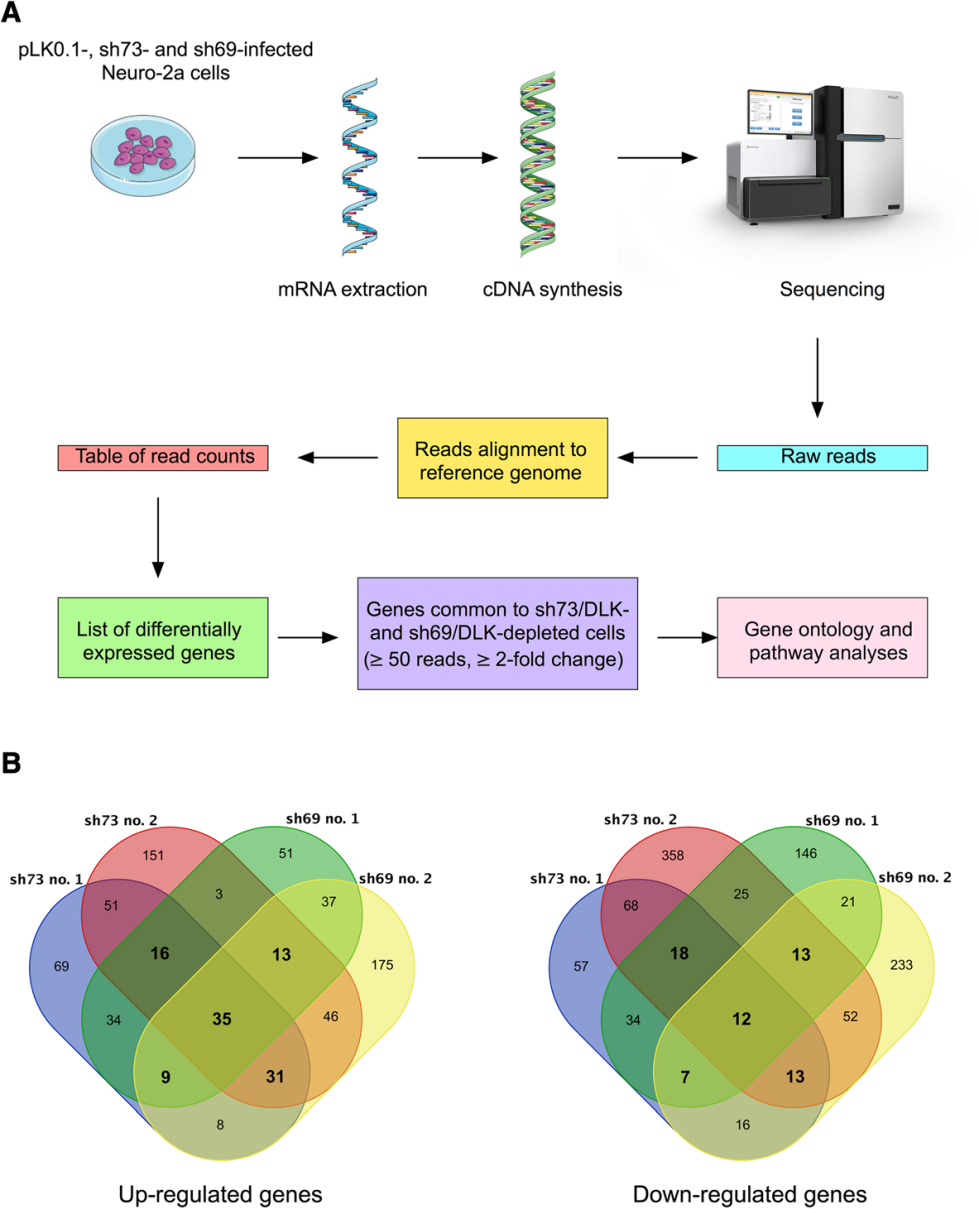
RNA-seq profiling of Neuro-2a cells after DLK depletion. **a** Scheme of the experimental design to identify differentially expressed genes (DEGs) in Neuro-2a cells after DLK depletion. **b** Venn diagrams showing the numbers of genes increased or decreased by two-fold or more in sh73/DLK- and sh69/DLK-depleted cells compared to control cells. Numbers in bold represent genes common to at least three of the four samples of DLK-depleted cells

To identify downstream effector genes of the DLK signaling pathway, we carried out RNA-seq experiments on both control and DLK-depleted Neuro-2a cells after differentiation (Fig. 3a). In order to improve downstream statistical analyses and minimize off-target effects of RNA interference, RNA-seq was performed in parallel on the two DLK-depleted cell lines described above and on two biological replicates for each condition. cDNA librairies constructed with mRNA isolated from differentiated control and DLK-depleted cells were subjected to 50 bp paired-end sequencing multiplexed in two lanes on an Illumina HiSeq system, which generated approximately 80–137 million reads per sample (Additional file 2: Table S2). After trimming, the high quality reads were aligned to the mouse reference genome (mm10) using the TopHat software [33]. Approximately 97 % of the high quality reads from each sample aligned to the reference genome (Additional file 2: Table S2), thus validating the quality and specificity of our transcriptome approach.

The RNA-seq data were subsequently analyzed using the HTSeq [36], DESeq [37] and edgeR [38] softwares to identify differentially expressed genes (DEGs) between control and DLK-depleted cells. As an additional filter on these data, we excluded from the analyses all genes with less than 50 reads on average per condition and with less than two-fold changes in expression as compared to control. To further narrow our candidate gene list, we then focused on up-regulated and down-regulated genes common to at least three of the four samples of sh73/DLK- and sh69/DLK-depleted cells (Fig. 3b). According to these parameters, 104 genes were found to be up-regulated after DLK depletion compared to control cells (Additional file 3: Table S3), whereas 63 were down-regulated (Additional file 4: Table S4).

This list of induced and repressed genes was next imported into the web-based bioinformatics tool Database for Annotation, Visualization and Integrated Discovery (DAVID) [41] to identify the most statistically significant functional annotation terms associated with them. Table 1 displays the top ten SP-PIR keywords enriched in our gene list with unadjusted and adjusted (Benjamini) P values. According to these data, more than 30 % of the DEGs show a significant association with terms related to signal-, glycoprotein- or membrane-cellular events. In addition, analysis of gene ontology (GO) terms in the biological process category for these up- and down-regulated genes revealed enrichment for genes associated with system development, protein phosphorylation and cell adhesion (Table 2). Among those up-regulated or down-regulated genes after DLK depletion, several were well-established regulators of biological processes related to neuron generation, development and differentiation, thus supporting the importance of DLK in nervous system structure and function. Included in this subset of DEGs are, for example, *Epha7*, *Nfasc* and *Nrp1*, which undergo up-regulation by two-fold or more in DLK-depleted cells (Additional file 3: Table S3), and *Anks1b*, *Rnd1* and *Sema6b* as genes whose expression decreased after DLK loss (Additional file 4: Table S4).

**Table 1:** SP-PIR keyword terms associated with dysregulated genes in DLK-depleted cells

Term	Count	%	*P*-Value	Benjamini
Signal	54	32,9	5,40E-09	1,20E-06
Glycoprotein	58	35,4	8,50E-08	9,50E-06
Extracellular matrix	11	6,7	9,60E-06	7,10E-04
Disulfide bond	41	25	9,70E-06	5,40E-04
Secreted	25	15,2	4,60E-04	2,00E-02
Cell adhesion	11	6,7	1,10E-03	4,00E-02
Membrane	58	35,4	1,80E-02	4,40E-01
Phosphotransferase	5	3	1,80E-02	4,10E-01
Smooth muscle	2	1,2	3,20E-02	5,50E-01
ATP	5	3	3,50E-02	5,40E-01

**Table 2:** Gene ontology terms associated with dysregulated genes in DLK-depleted cells

Term	Count	%	*P*-Value	Benjamini
Protein amino acid phosphorylation	15	9,1	3,70E-04	3,30E-01
System development	29	17,7	1,10E-03	4,50E-01
Phosphorylation	15	9,1	1,20E-03	3,40E-01
Enzyme linked receptor protein signaling pathway	9	5,5	1,20E-03	2,70E-01
Anatomical structure development	29	17,7	3,20E-03	4,90E-01
Cell adhesion	12	7,3	3,80E-03	4,90E-01
Biological adhesion	12	7,3	3,90E-03	4,50E-01
Extracellular structure organization	6	3,7	5,80E-03	5,40E-01
Phosphorus metabolic process	15	9,1	6,30E-03	5,30E-01
Phosphate metabolic process	15	9,1	6,30E-03	5,30E-01

**Fig. 4:**
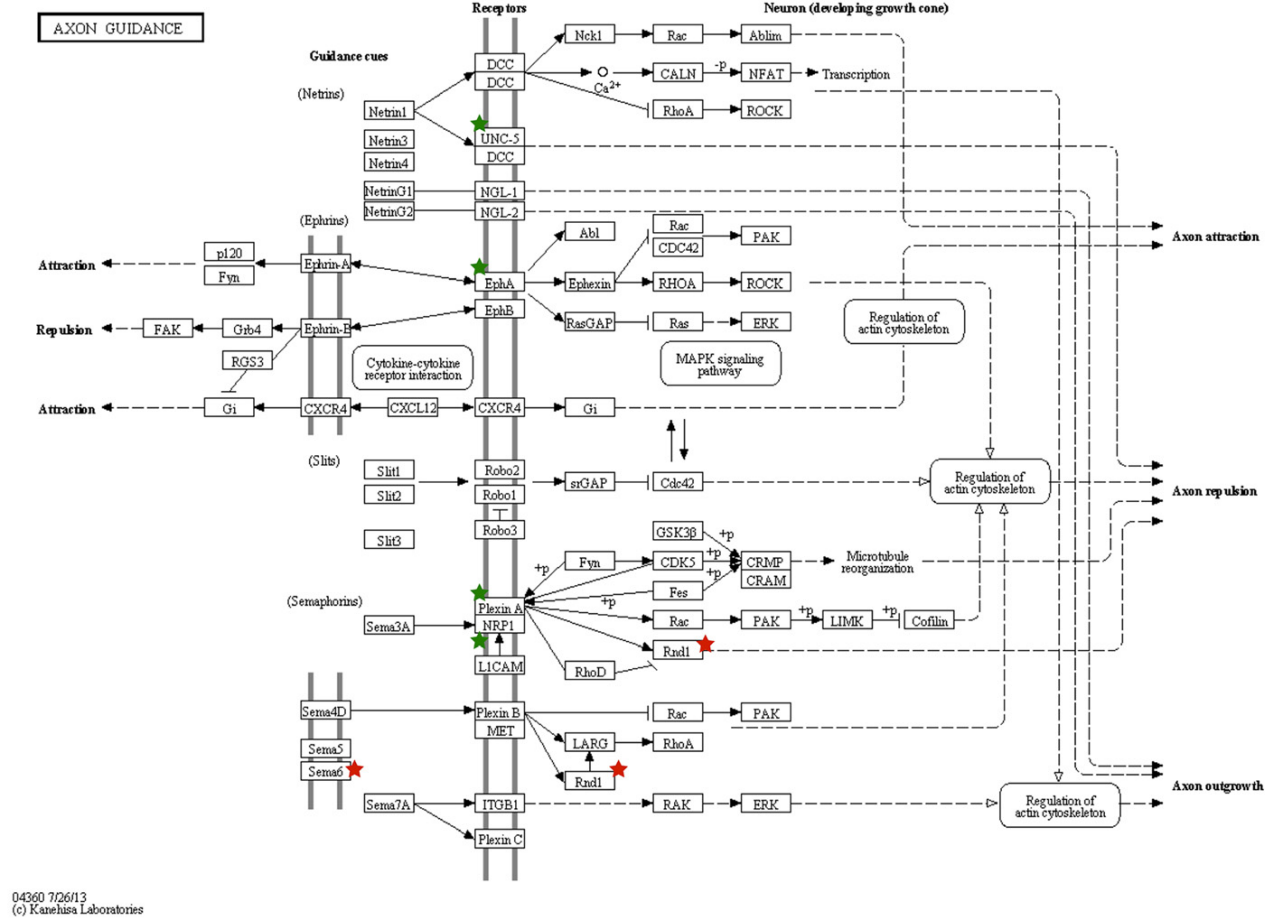
KEGG pathway of axon guidance showing the genes that are either up- (green stars) or down-regulated (red stars) in sh73/DLK- and sh69/DLK-depleted cells

A complementary analysis using KEGG pathway mapping also revealed that the genes dysregulated in DLK-depleted cells were enriched for functions related to axon guidance, extracellular matrix-receptor interaction and focal adhesion (Table 3). Because axon guidance was the top enriched pathway based on the *P*-value, we decided to focus our experimental efforts on this group of genes for further characterization. These putative DLK-regulated genes fell into three distinct functional categories of axon guidance molecules, namely membrane-bound ligand (*Sema6b*), cell surface receptors (*Epha7*, *Plxna4*, *Nrp1*, *Unc5a*) and GTP-binding protein (*Rnd1*) (Fig. 4). Table 4 indicates that *Epha7*, *Nrp1*, *Plxna4* and *Unc5a* were up-regulated in DLK-depleted cells, whereas *Rnd1* and *Sema6b* were down-regulated.

**Table 3:** KEGG pathway terms associated with dysregulated genes in DLK-depleted cells

Term	Count	%	Gene symbols	*P*-Value	Benjamini
Axon guidance	6	3,7	*Epha7, Rnd1, Nrp1, Plxna4, Sema6b, Unc5a*	3,70E-03	2,50E-01
ECM-receptor interaction	5	3	*Itgb3, Itgb4, Lama2, Tnr, Thbs3*	4,20E-03	1,50E-01
Focal adhesion	7	4,3	*Hgf, Itgb3, Itgb4, Lama2, Pdgfra, Tnr, Thbs3*	4,60E-03	1,10E-01
Arrhythmogenic right ventricular cardiomyopathy (ARVC)	4	2,4	*Des, Itgb3, Itgb4, Lama2*	2,20E-02	3,50E-01
Hypertrophic cardiomyopathy (HCM)	4	2,4	*Des, Itgb3, Itgb4, Lama2*	2,90E-02	3,70E-01

**Table 4:** Fold change of the axon guidance genes dysregulated in DLK-depleted cells relative to control cells

Ensembl gene id	Symbol	Description	Log2 fold-change				Regulation
			sh73 no. 1	sh73 no. 2	sh69 no. 1	sh69 no. 2	
ENSMUSG00000028289	*Epha7*	Eph receptor A7	1.657	1.741	1.387	1.595	Up
ENSMUSG00000054855	*Rnd1*	Rho family GTPase 1	−1.436	−1.463	−1.112	*−0.529*	Down
ENSMUSG00000025810	*Nrp1*	Neuropilin 1	1.14	1.304	*0.72*	1.383	Up
ENSMUSG00000029765	*Plxna4*	Plexin A4	1.151	*0.699*	1.522	1.367	Up
ENSMUSG00000001227	*Sema6B*	Semaphorin 6B	−1.055	−1.797	*−0.866*	−1.780	Down
ENSMUSG00000025876	*Unc5a*	Unc-5 homolog A	1.283	1.451	*0.977*	1.127	Up

Numbers in italics represent change in gene expression that were less than two-fold

### Validation of RNA-seq data by qRT-PCR analysis and immunoblotting

**Fig. 5:**
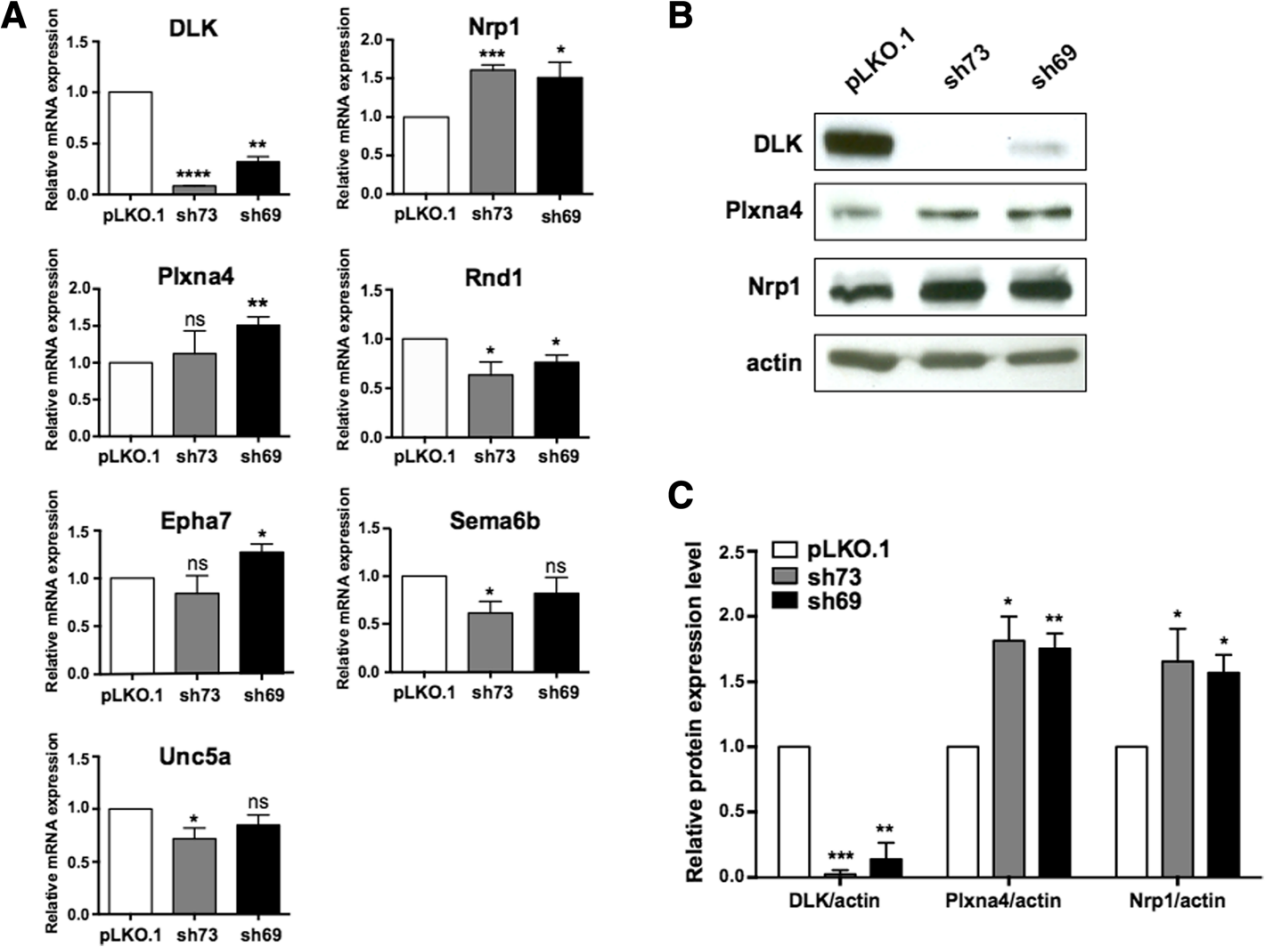
Validation of RNA-seq data by qRT-PCR and Western blot analyses. **a** The relative mRNA level of DLK and axon guidance genes in infected cells was analyzed by qRT-PCR, normalized to three housekeeping genes and calculated with the ΔΔ*C*_*T*_ method. The value of mRNA expression for each gene in control cells (pLKO.1) was arbitrarily set to 1. Data are the mean ± SEM (error bars) from three independent experiments carried out in triplicate. *, *p* < 0.05; **, *p* < 0.01; ***, *p* < 0.001; ****, *p* < 0.0001; ns, *p* > 0.05. **b** Representative Western blots showing levels of DLK, Plxna4, Nrp1 and actin in control and DLK-depleted Neuro-2a cells. **c** Quantitative densitometric measurements of DLK, Plxna4 and Nrp1 protein levels in infected cells. Results are normalized to actin level in control cells, which were set to 1, and represent mean ± SEM (error bars) from three independent experiments. *, *p* < 0.05; **, *p* < 0.01; ***, *p* < 0.001

The six axon guidance genes identified by RNA-seq as being either up-regulated or down-regulated by DLK knockdown were validated by qRT-PCR. As shown in Fig. 5a, qRT-PCR analysis of cells expressing either the sh73 or sh69 lentiviral construct confirmed the results obtained by RNA-seq for most of the selected genes, indicating the reliability of our transcriptomic data. One exception was *Unc5a*, which was found by RNA-seq and qRT-PCR analyses to be regulated in opposite directions. For genes such as *Nrp1* and *Rnd1*, our qRT-PCR results were correlated with the RNA-seq results for both DLK shRNA constructs, whereas for the other genes tested, including *Sema6b*, *Epha7* and *Plxna4*, a correlation was seen only with either the sh73 or the sh69 lentiviral vector. The reason behind this result is not clear, but it most likely relates to the fact that the residual amount of DLK in cells after knockdown varies between the two constructs and between experiments, which could consequently lead to differential effects on gene expression.

As a complementary approach, we also analyzed whether the differences in transcript abundance between control and DLK-depleted cells translated into protein level changes for two of the identified axon guidance genes, namely *Plxna4* and *Nrp1*. These two genes were of particular interest because they encode cell-surface receptors that act in complex to mediate repulsive responses of axons to class-3 semaphorins in most neurons [45]. Moreover, they have been recognized as important regulators of neuronal migration [46], a process impaired in DLK knockout mouse brain [11]. In accordance with RNA-seq and qRT-PCR data, immunoblot analysis showed that protein levels of Plxna4 and Nrp1 were significantly increased by 1.5- to 2-fold in Neuro-2a cells after DLK depletion compared with control (Fig. 5b and c). Taken together, these experiments confirm that DLK depletion has an effect on Plxna4 and Nrp1 expression in neuronal cells at both the transcriptional and translational levels, and shed light for the first time on the mechanisms by which DLK signalling contributes to nervous system development and function.

## Discussion

Recent exciting findings based on experiments carried out in vivo and in vitro have highlighted the potential role of DLK in nervous system assembly, maintenance and repair. These studies have indeed demonstrated the requirement for DLK in various aspects of neuronal cell physiology, ranging from migration, axon growth and apoptosis during development to nerve regeneration and degeneration in the adult (for a review, see [47]). One of the fundamental issues remaining to be solved in this context is elucidation of the mechanisms by which DLK regulates such a vast range of biological responses. In an attempt to gain insight into DLK’s mode of action, we have examined the global changes that occur in the transcriptome of differentiated Neuro-2a cells after knockdown of DLK by RNA interference. Our results demonstrate that DLK depletion leads to a decrease or an increase in the levels of numerous mRNAs, indicating that DLK contributes to both positive and negative regulation of gene expression even under basal conditions. At this point, it is not known whether these changes in gene expression are a cause or a consequence of DLK depletion. However, we assume that such a response is probably JNK-dependent because knockdown of DLK in Neuro-2a cells impairs JNK basal activity and JNK-mediated phosphorylation of c-Jun, a component of the AP-1 transcription factor (Fig. 1). This assumption is also supported by the studies of Hirai et al. (2006) who showed that JNK activity and phosphorylation of JNK substrates, including c-Jun, are significantly reduced in DLK^−/−^ mouse embryonic brain. Previous reports found that JNK both positively and negatively regulates gene expression by phosphorylating DNA-bound proteins, such as transcription factors and histones, as well as by binding directly to transcriptionally active gene promoters [48, 49].

Interestingly, decrease of DLK expression in Neuro-2a cells resulted in transcriptional dysregulation of a subset of genes involved in neuronal function, such as migration, differentiation, axonogenesis, and axon guidance, an observation that supports its critical role in nervous system development (Additional file 3: Table S3, Additional file 4: Table S4). To our knowledge, none of these genes have been previously identified as potential targets of DLK. In a recent work using a microarray approach, Watkins et al. (2013) showed that DLK is required for expression of proapoptotic- and regeneration-related genes in retinal ganglion cells (RGCs) after optic nerve crush. Consistent with this finding, it has also been reported that RGCs isolated from mice containing a floxed allele of DLK displayed resistance to death induced by axonal injury, together with a concomitant decrease in JNK phosphorylation and c-Jun expression [17]. The basis for the difference in target gene specificity between Neuro-2a cells and RGCs is unclear, but could reflect cell type or cell context variability. Another possible explanation might be that the DLK-dependent transcriptional program varies between basal and stress-stimulated conditions, which inevitably lead to different cellular responses.

As stated above, the current results highlight for the first time the involvement of DLK in expression of axon guidance genes. This family of genes encodes proteins that act as attractants or repellents for axons, thereby guiding them towards or away from a specific region [23, 50]. The growth cone, a sensory structure located at the tip of extending axons, expresses receptors that recognize these guidance cues and trigger intracellular signaling cascades, resulting in extensive cytoskeletal rearrangement and subsequent axon steering [51]. Different families of axon guidance cues and receptors have been identified, including ephrins and Eph receptors, semaphorins and plexin and neuropilin receptors, Slits and Robo receptors, Netrins, DCC and Unc5 receptors as well as RGM and noegenin receptors [23]. Axon guidance proteins have been shown to be involved in various aspects of neural circuit development (e.g., growth, guidance, bundling, fasciculation and pruning of axons, and synaptogenesis), and in the control of synaptic plasticity in adults [52]. Emerging data from human and animal models also implicate axon guidance molecules in neurological disorders and axon regeneration after injury [24, 53–55]. In our RNA-seq data, four axon guidance genes, including *Epha7*, *Nrp1*, *Plxna4* and *Unc5*a, showed an increase in expression level after DLK depletion, whereas two, *Rnd1* and *Sema6b*, were down-regulated (Additional file 3: Table S3 and Additional file 4: Table S4). qRT-PCR confirmed that these mRNA changes were significant for *Epha7*, *Nrp1*, *Plxna4, Rnd1* and *Sema6b* (Fig. 5). Because there was no correlation between the RNA-seq and qRT-PCR results for *Unc5a*, we currently cannot draw any conclusion for this gene. A part of our findings was further validated by Western blot analysis, which shows that Nrp1 and Plxna4 protein levels were significantly induced in DLK-depleted cells (Fig. 5), thus demonstrating the ability of DLK to exert a repressing effect on Nrp1 and Plxna4 expression. Because Nrp1 and Plxna4 cooperate to mediate signaling in response to semaphorin 3A (Sema3A), a repulsive guidance cue for axons in most neurons [25], it is tempting to speculate that an increase in their expression, such as the one described here, could contribute to the defects of axonal growth previously described in DLK null mouse embryos and DLK-deficient cultured cortical neurons [11, 44]. This hypothesis is fully in line with results demonstrating that ectopic expression of Nrp1 or Plxna4 in neuronal cell types that are insensitive to Sema3a caused axon repulsion and growth cone collapse in response to Sema3A [45, 56]. The identification of *Epha7* and *Rnd1* as up- and down-regulated genes, respectively, in DLK-depleted cells is also of great interest in regard to the contribution of DLK to neuron projection development. Indeed, a recent study revealed that Epha7, which generally transduces repulsive signals [57], impairs dendrite formation when overexpressed in cortical neurons [58], while knockdown of Rnd1 in hippocampal neurons suppressed axon extension [59]. Finally, for *Sema6b*, whose expression decreased upon DLK knockdown in Neuro-2a cells, results obtained from loss-of-function experiments demonstrated its requirement for proper projection of hippocampal granule cell axons [60]. Taken together, these data are supportive of a model whereby DLK promotes axonal growth during neural development by regulating, at least in part, the expression of axon guidance genes. It is presently not known how DLK would modulate axon guidance molecule abundance in neurons, but one possible mechanism is control of synthesis, phosphorylation and/or activity of the transcription factors that regulate them. Work carried out over the past several years has established that transcriptional control of axon guidance cues and receptors is crucial to allow precise pathfinding decisions of neuronal growth cones [61, 62].

Although further work will be required to investigate the biological consequences of dysregulated axon guidance gene expression in DLK-depleted Neuro-2a cells, we anticipate as a potential outcome an alteration of the cytoskeleton. This prediction is supported by the fact that axon guidance cues and their receptors modulate actin and microtubule dynamics in the growth cone via activation of downstream signaling molecules, such as protein kinases, small GTPases and cytoskeleton-associated proteins [50, 63, 64]. As illustrated by studies in different systems, remodeling of the cytoskeleton is critically important for attraction, repulsion, growth cone collapse or axon extension [51]. Interestingly, a functional link between DLK and cytoskeletal regulation has been recently suggested. In this study, Hirai et al. (2011) demonstrated by RNA interference that knockdown of DLK in cultured mouse embryonic neurons caused defects in axon formation, whereas concomitant treatment of cells with the microtubule-stabilizing drug taxol antagonized this response. These results implicate DLK as a key regulator of microtubule stabilization, a required event for axon formation [65, 66]. Consistent with an active role for DLK in microtubule dynamics, DLK gene disruption in mice resulted in reduced phosphorylation of the microtubule-stabilizing proteins Doublecortin and MAP2c [11]. In light of these data and the results from the present study, it is conceivable to suggest that DLK regulation of axon growth in neurons may depend, at least in part, on cytoskeletal reorganization mediated either directly via phosphorylation of microtubule-associated proteins or indirectly through modulation of axon guidance gene expression.

## Conclusions

We found that DLK plays an important role in regulating expression of genes recognized for their contribution in nervous system development and function. Future studies will be dedicated to understand how DLK influences the expression of *Nrp1*, *Plxna4*, *Epha7*, *Rnd*1 and *Sema6b* as well as the consequences of their up- or down-regulation on axon growth. Further experiments using the DLK knockout mice will also be required to assess the in vivo relevance of our in vitro findings.

## Abbreviations

DLK, dual leucine zipper kinase; Epha7, eph receptor A7; ERK, extracellular signal-regulated kinase; JNK, c-Jun N-terminal kinase; Nrp1, neuropilin 1; Plxna4, plexin A4; Rnd1, Rho family GTPase 1; Sema6b, semaphorin 6B; Unc5a, Unc-5 homolog A

## Additional files

Additional file 1: Table S1. Primers used in this study. (PDF 60 kb) Additional file 2: Table S2. Transcriptome read statistics. (PDF 49 kb) Additional file 3: Table S3. Genes up-regulated by two-fold or more in DLK-depleted cells. (PDF 103 kb) Additional file 4: Table S4. Genes down-regulated by two-fold or more in DLK-depleted cells. (PDF 94 kb)
