# Supplementary material for: Dual leucine zipper kinase regulates expression of axon guidance genes in mouse neuronal cells
Source: Neural Dev. 2016 Jul 28;11:13. doi: 10.1186/s13064-016-0068-8 (PMC4965899; doi:10.1186/s13064-016-0068-8)
Supplement: Additional file 1: Table S1. — Primers used in this study. (PDF 60 kb) [file 13064_2016_68_MOESM1_ESM.pdf]

Table S1. Primers used in this study

Genes	Forward primer	Reverse primer
Map3k12	ATCATCTGGGGTGTGGGAAGCA	GATCTGTCGGAATGATGGGCGAT
Epha7	AGCTCCTTCTGATCCACCATACGT	GTGACATCGTTTCTTCCCCCGT
Rnd1	TCAGATGCGGTATTGCTGTGCT	ACGTGTGCTGGGACAGTAGTCT
Nrp1	ACATGGGGCAGGGTTTTCCATC	AGCTGTTGGGGTATTTTTCAGGGAA
Plxna4	GCTGGTTGGGGAGAAACCATGC	GGGCTGTCTGGGGCGATGTATA
Sema6B	TTCCTGTTGCCCGTTGCCAG	GGTCTGAGGAAGATGCAGGAGC
Unc5a	CCGCAAGAAGGAAGGACTGGAC	GTAGGTGGTCGTGGTGGTGCT
Pum1	TGCCAGTCTCTTCCAGCAGCA	TGATTTGGGGTCAAAGGACGTTGG
Sdha	CGGCTTTCACTTCTCTGTTGGTGA	AAAGGCCAAATGCAGCTCGCAA
Txnl4b	CCCTCTACCGTATTTTTCTTCAATGGGC	AGTTTTCCCCTCATCGCTCCCC
