# Supplementary material for: Dual leucine zipper kinase regulates expression of axon guidance genes in mouse neuronal cells
Source: Neural Dev. 2016 Jul 28;11:13. doi: 10.1186/s13064-016-0068-8 (PMC4965899; doi:10.1186/s13064-016-0068-8)
Supplement: Additional file 2: Table S2. — Transcriptome read statistics. (PDF 49 kb) [file 13064_2016_68_MOESM2_ESM.pdf]

Table S2. Transcriptome read statistics

Sample	No. of raw reads	No. of high-quality reads	% of high-quality reads	No. of aligned reads	% of aligned reads
pLK0.1 no. 1	137 446 284	125 748 138	91.5	123 001 772	97.8
pLK0.1 no. 2	122 866 332	111 260 674	90.6	109 116 527	98.1
sh73 no. 1	113 242 930	100 427 570	88.7	96 965 350	96.6
sh73 no. 2	136 068 740	126 035 908	91.3	122 218 239	97.0
sh69 no. 1	127 978 034	114 082 758	89.1	111 713 191	97.9
sh69 no. 2	80 794 220	75 337 030	93.2	73 693 654	97.8
