# Supplementary material for: Dual leucine zipper kinase regulates expression of axon guidance genes in mouse neuronal cells
Source: Neural Dev. 2016 Jul 28;11:13. doi: 10.1186/s13064-016-0068-8 (PMC4965899; doi:10.1186/s13064-016-0068-8)
Supplement: Additional file 3: Table S3. — Genes up-regulated by two-fold or more in DLK-depleted cells. (PDF 103 kb) [file 13064_2016_68_MOESM3_ESM.pdf]

Table S3. Genes up-regulated by two-fold or more in DLK-depleted cells

| Ensembl Gene ID | Gene Symbol | Gene description |
|---|---|---|
| ENSMUSG00000052334 | 1700024B05Rik | RIKEN cDNA 1700024B05 gene |
| ENSMUSG00000057880 | *Abat* | 4-aminobutyrate aminotransferase |
| ENSMUSG00000035783 | *Acta2* | actin, alpha 2, smooth muscle, aorta |
| ENSMUSG00000054555 | *Adam12* | a disintegrin and metallopeptidase domain 12 (meltrin alpha) |
| ENSMUSG00000028782 | *Adgrb2* | brain-specific angiogenesis inhibitor 2 |
| ENSMUSG00000033569 | *Adgrb3* | brain-specific angiogenesis inhibitor 3 |
| ENSMUSG00000015452 | *Ager* | advanced glycosylation end product-specific receptor |
| ENSMUSG00000021057 | *Akap5* | A kinase (PRKA) anchor protein 5 |
| ENSMUSG00000029455 | *Aldh2* | aldehyde dehydrogenase 2, mitochondrial |
| ENSMUSG00000022265 | *Ank* | progressive ankylosis |
| ENSMUSG00000079105 | *C7* | complement component 7 |
| ENSMUSG00000074715 | *Ccl28* | chemokine (C-C motif) ligand 28 |
| ENSMUSG00000032218 | *Ccnb2* | cyclin B2 |
| ENSMUSG00000026605 | *Cenpf* | centromere protein F |
| ENSMUSG00000097164 | *Cep83os* | centrosomal protein 83, opposite strand |
| ENSMUSG00000027350 | *Chgb* | chromogranin B |
| ENSMUSG00000022371 | *Col14a1* | collagen, type XIV, alpha 1 |
| ENSMUSG00000032291 | *Crabp1* | cellular retinoic acid binding protein I |
| ENSMUSG00000000416 | *Cttnbp2* | cortactin binding protein 2 |
| ENSMUSG00000035910 | *Dcdc2a* | doublecortin domain containing 2a |
| ENSMUSG00000059213 | *Ddn* | dendrin |
| ENSMUSG00000015377 | *Dennd6b* | family with sequence similarity 116, member B |
| ENSMUSG00000069814 | E130309D14Rik | RIKEN cDNA E130309D14 gene |
| ENSMUSG00000037868 | *Egr2* | early growth response 2 |
| ENSMUSG00000036941 | *Elac1* | elaC homolog 1 (E. coli) |
| ENSMUSG00000029675 | *Eln* | elastin |
| ENSMUSG00000034164 | *Emid1* | EMI domain containing 1 |
| ENSMUSG00000028289 | *Epha7* | Eph receptor A7 |
| ENSMUSG00000039157 | *Fam102a* | family with sequence similarity 102, member A |
| ENSMUSG00000019779 | *Frk* | fyn-related kinase |
| ENSMUSG00000036264 | *Fstl4* | follistatin-like 4 |
| ENSMUSG00000050288 | *Fzd2* | frizzled homolog 2 (Drosophila) |
| ENSMUSG00000006344 | *Ggt5* | gamma-glutamyltransferase 5 |
| ENSMUSG00000098178 | *Gm42418* | predicted gene, 42418 |
| ENSMUSG00000039720 | *Got1l1* | glutamic-oxaloacetic transaminase 1-like 1 |
| ENSMUSG00000040133 | *Gpr176* | G protein-coupled receptor 176 |
| ENSMUSG00000028864 | *Hgf* | hepatocyte growth factor |
| ENSMUSG00000018102 | *Hist1h2bc* | histone cluster 1, H2bc |

| | | |
|---|---|---|
| ENSMUSG00000050936 | *Hist2h2bb* | histone cluster 2, H2bb |
| ENSMUSG00000022485 | *Hoxc5* | homeo box C5 |
| ENSMUSG00000039059 | *Hrh3* | histamine receptor H3 |
| ENSMUSG00000032269 | *Htr3a* | 5-hydroxytryptamine (serotonin) receptor 3A |
| ENSMUSG00000008590 | *Htr3b* | 5-hydroxytryptamine (serotonin) receptor 3B |
| ENSMUSG00000021379 | *Id4* | inhibitor of DNA binding 4 |
| ENSMUSG00000032394 | *Igdcc3* | immunoglobulin superfamily, DCC subclass, member 3 |
| ENSMUSG00000029814 | *Igf2bp3* | insulin-like growth factor 2 mRNA binding protein 3 |
| ENSMUSG00000026185 | *Igfbp5* | insulin-like growth factor binding protein 5 |
| ENSMUSG00000020689 | *Itgb3* | integrin beta 3 |
| ENSMUSG00000020758 | *Itgb4* | integrin beta 4 |
| ENSMUSG00000058975 | *Kcnc1* | potassium voltage gated channel, Shaw-related subfamily, member 1 |
| ENSMUSG00000059742 | *Kcnh7* | potassium voltage-gated channel, subfamily H (eag-related), member 7 |
| ENSMUSG00000019899 | *Lama2* | laminin, alpha 2 |
| ENSMUSG00000060594 | *Layn* | layilin |
| ENSMUSG00000026443 | *Lrrn2* | leucine rich repeat protein 2, neuronal |
| ENSMUSG00000034041 | *Lyl1* | lymphoblastomic leukemia 1 |
| ENSMUSG00000051098 | *Mblac2* | metallo-beta-lactamase domain containing 2 |
| ENSMUSG00000021596 | *Mctp1* | multiple C2 domains, transmembrane 1 |
| ENSMUSG00000024593 | *Megf10* | multiple EGF-like-domains 10 |
| ENSMUSG00000033752 | *Mnd1* | meiotic nuclear divisions 1 homolog |
| ENSMUSG00000029376 | *Mthfd2l* | methylenetetrahydrofolate dehydrogenase (NADP+ dependent) 2-like |
| ENSMUSG00000023341 | *Mx2* | myxovirus (influenza virus) resistance 2 |
| ENSMUSG00000038670 | *Mybpc2* | myosin binding protein C, fast-type |
| ENSMUSG00000041132 | *N4bp2l1* | NEDD4 binding protein 2-like 1 |
| ENSMUSG00000026442 | *Nfasc* | neurofascin |
| ENSMUSG00000067786 | *Nnat* | neuronatin |
| ENSMUSG00000028469 | *Npr2* | natriuretic peptide receptor 2 |
| ENSMUSG00000029819 | *Npy* | neuropeptide Y |
| ENSMUSG00000025810 | *Nrp1* | neuropilin 1 |
| ENSMUSG00000027939 | *Nup210l* | nucleoporin 210-like |
| ENSMUSG00000038463 | *Olfml2b* | olfactomedin-like 2B |
| ENSMUSG00000062257 | *Opcml* | opioid binding protein/cell adhesion molecule-like |
| ENSMUSG00000026525 | *Opn3* | opsin 3 |
| ENSMUSG00000041119 | *Pde9a* | phosphodiesterase 9A |
| ENSMUSG00000029231 | *Pdgfra* | platelet derived growth factor receptor, alpha polypeptide |
| ENSMUSG00000022197 | *Pdzd2* | PDZ domain containing 2 |

| | | |
|---|---|---|
| ENSMUSG00000036834 | *Plch1* | phospholipase C, eta 1 |
| ENSMUSG00000035835 | *Plppr3* | cDNA sequence BC005764 |
| ENSMUSG00000033342 | *Plppr5* | RIKEN cDNA 4833424O15 gene |
| ENSMUSG00000029765 | *Plxna4* | plexin A4 |
| ENSMUSG00000036158 | *Prickle1* | prickle like 1 (Drosophila) |
| ENSMUSG00000029838 | *Ptn* | pleiotrophin |
| ENSMUSG00000043587 | *Pxylp1* | acid phosphatase-like 2 |
| ENSMUSG00000022159 | *Rab2b* | RAB2B, member RAS oncogene family |
| ENSMUSG00000088088 | *Rmrp* | RNA component of mitochondrial RNAase P |
| ENSMUSG00000028278 | *Rragd* | Ras-related GTP binding D |
| ENSMUSG00000045287 | *Rtn4rl1* | reticulon 4 receptor-like 1 |
| ENSMUSG00000021703 | *Serinc5* | serine incorporator 5 |
| ENSMUSG00000000632 | *Sez6* | seizure related gene 6 |
| ENSMUSG00000037112 | *Sik2* | salt inducible kinase 2 |
| ENSMUSG00000033147 | *Slc22a15* | Solute carrier family 22 member 15 |
| ENSMUSG00000046329 | *Slc25a23* | solute carrier family 25 (mitochondrial carrier; phosphate carrier), member 23 |
| ENSMUSG00000029309 | *Sparcl1* | SPARC-like 1 |
| ENSMUSG00000035493 | *Tgfbi* | transforming growth factor, beta induced |
| ENSMUSG00000028047 | *Thbs3* | thrombospondin 3 |
| ENSMUSG00000043843 | *Tmem145* | transmembrane protein 145 |
| ENSMUSG00000015829 | *Tnr* | tenascin R |
| ENSMUSG00000036964 | *Trim17* | tripartite motif-containing 17 |
| ENSMUSG00000021541 | *Trpc7* | transient receptor potential cation channel, subfamily C, member 7 |
| ENSMUSG00000058254 | *Tspan7* | tetraspanin 7 |
| ENSMUSG00000025876 | *Unc5a* | unc-5 homolog A (C. elegans) |
| ENSMUSG00000021994 | *Wnt5a* | wingless-related MMTV integration site 5A |
| ENSMUSG00000036699 | *Zcchc12* | zinc finger, CCHC domain containing 12 |
| ENSMUSG00000045639 | *Zfp629* | zinc finger protein 629 |
| ENSMUSG00000063894 | *Zkscan8* | zinc finger protein 192 |

Green background denotes genes related to neuronal functions as defined by gene ontology annotation and/or KEGG analysis.
