# Supplementary material for: Dual leucine zipper kinase regulates expression of axon guidance genes in mouse neuronal cells
Source: Neural Dev. 2016 Jul 28;11:13. doi: 10.1186/s13064-016-0068-8 (PMC4965899; doi:10.1186/s13064-016-0068-8)
Supplement: Additional file 4: Table S4. — Genes down-regulated by two-fold or more in DLK-depleted cells. (PDF 94 kb) [file 13064_2016_68_MOESM4_ESM.pdf]

Table S4. Genes down-regulated by two-fold or more in DLK-depleted cells

Ensembl Gene ID	Gene Symbol	Gene description
ENSMUSG00000096232	0610012G03Rik	RIKEN cDNA 0610012G03 gene
ENSMUSG00000078607	1810010H24Rik	RIKEN cDNA 1810010H24 gene
ENSMUSG00000097207	6030443J06Rik	RIKEN cDNA 6030443J06 gene
ENSMUSG00000097591	A330032B11Rik	RIKEN cDNA A330032B11 gene
ENSMUSG00000021226	*Acot2*	acyl-CoA thioesterase 2
ENSMUSG00000046169	*Adamts6*	a disintegrin-like and metallopeptidase (reprolysin type) with thrombospondin type 1 motif, 6
ENSMUSG00000023918	*Adgrf4*	G protein-coupled receptor 115
ENSMUSG00000072812	*Ahnak2*	AHNAK nucleoprotein 2; similar to Unknown (protein for IMAGE:3599271)
ENSMUSG00000029762	*Akr1b8*	aldo-keto reductase family 1, member B8
ENSMUSG00000002661	*Alkbh7*	alkB, alkylation repair homolog 7 (E. coli)
ENSMUSG00000044037	*Als2cl*	ALS2 C-terminal like
ENSMUSG00000023047	*Amhr2*	anti-Mullerian hormone type 2 receptor
ENSMUSG00000038742	*Angptl6*	angiopoietin-like 6
ENSMUSG00000050914	*Ankrd37*	ankyrin repeat domain 37
ENSMUSG00000058589	*Anks1b*	ankyrin repeat and sterile alpha motif domain containing 1B
ENSMUSG00000030220	*Arhgdib*	Rho, GDP dissociation inhibitor (GDI) beta
ENSMUSG00000018821	*Avpi1*	arginine vasopressin-induced 1
ENSMUSG00000078566	*Bnip3*	predicted gene 14506; BCL2/adenovirus E1B interacting protein 3; predicted gene 6532; similar to E1B 19K/Bcl-2-binding protein homolog
ENSMUSG00000057897	*Camk2b*	calcium/calmodulin-dependent protein kinase II, beta
ENSMUSG00000039518	*Cdsn*	corneodesmosin; hypothetical protein LOC100043961
ENSMUSG00000004665	*Cnn2*	calponin 2
ENSMUSG00000037190	*Cyb561d2*	cytochrome b-561 domain containing 2
ENSMUSG00000044795	*Cyb5d1*	cytochrome b5 domain containing 1; predicted gene 6685
ENSMUSG00000026208	*Des*	desmin
ENSMUSG00000056069	*Fam105a*	family with sequence similarity 105, member A
ENSMUSG00000045761	*Fam179a*	family with sequence similarity 179, member A
ENSMUSG00000069911	*Fam196b*	predicted gene 6041
ENSMUSG00000033386	*Frrs1*	ferric-chelate reductase 1
ENSMUSG00000063001	*Gm9701*	predicted gene 9701
ENSMUSG00000027610	*Gss*	glutathione synthetase
ENSMUSG00000026864	*Hspa5*	heat shock protein 5

ENSMUSG00000042745	*Id1*	inhibitor of DNA binding 1
ENSMUSG00000033581	*Igf2bp2*	insulin-like growth factor 2 mRNA binding protein 2
ENSMUSG00000055675	*Kbtbd11*	kelch repeat and BTB (POZ) domain containing 11
ENSMUSG00000002908	*Kcnn1*	potassium intermediate/small conductance calcium-activated channel, subfamily N, member 1
ENSMUSG00000027412	*Lpin3*	lipin 3
ENSMUSG00000033207	*Mamdc2*	MAM domain containing 2
ENSMUSG00000023050	*Map3k12*	mitogen-activated protein kinase kinase kinase 12
ENSMUSG00000032508	*Myd88*	myeloid differentiation primary response gene 88
ENSMUSG00000005125	*Ndrg1*	N-myc downstream regulated gene 1
ENSMUSG00000026162	*Nhej1*	nonhomologous end-joining factor 1
ENSMUSG00000026946	*Nmi*	N-myc (and STAT) interactor
ENSMUSG00000031410	*Nxf7*	nuclear RNA export factor 7
ENSMUSG00000026698	*Pigc*	phosphatidylinositol glycan anchor biosynthesis, class C
ENSMUSG00000029334	*Prkg2*	protein kinase, cGMP-dependent, type II
ENSMUSG00000070371	*Prss36*	protease, serine, 36
ENSMUSG00000015090	*Ptgds*	prostaglandin D2 synthase (brain)
ENSMUSG00000028378	*Ptgr1*	prostaglandin reductase 1
ENSMUSG00000047250	*Ptgs1*	prostaglandin-endoperoxide synthase 1
ENSMUSG00000010362	*Rdm1*	RAD52 motif 1
ENSMUSG00000054855	*Rnd1*	Rho family GTPase 1
ENSMUSG00000072714	*Rpl21-ps4*	ribosomal protein L21, pseudogene 4
ENSMUSG00000051169	*Rpusd3*	RNA pseudouridylate synthase domain containing 3
ENSMUSG00000001227	*Sema6b*	sema domain, transmembrane domain (TM), and cytoplasmic domain, (semaphorin) 6B
ENSMUSG00000037411	*Serpine1*	serine (or cysteine) peptidase inhibitor, clade E, member 1
ENSMUSG00000015112	*Slc25a13*	solute carrier family 25 (mitochondrial carrier, adenine nucleotide translocator), member 13
ENSMUSG00000029699	*Ssc4d*	scavenger receptor cysteine rich domain containing, group B (4 domains)
ENSMUSG00000034947	*Tmem106a*	transmembrane protein 106A
ENSMUSG00000032715	*Trib3*	tribbles homolog 3 (Drosophila)
ENSMUSG00000056832	*Ttc26*	tetratricopeptide repeat domain 26
ENSMUSG00000020407	*Upp1*	uridine phosphorylase 1
ENSMUSG00000060862	*Zbtb40*	similar to zinc finger and BTB domain containing 40; zinc finger and BTB domain containing 40

ENSMUSG00000059842	*Zfp341*	zinc finger protein 341

Red background denotes genes related to neuronal functions as defined by gene ontology annotation and/or KEGG analysis.
